# Involvement of MLPK Pathway in Intraspecies Unilateral Incompatibility Regulated by a Single Locus With Stigma and Pollen Factors

**DOI:** 10.1534/g3.113.005892

**Published:** 2013-04-01

**Authors:** Yoshinobu Takada, Takahiro Sato, Go Suzuki, Hiroshi Shiba, Seiji Takayama, Masao Watanabe

**Affiliations:** * Graduate School of Life Sciences, Tohoku University, Sendai 980-8577, Japan; † Division of Natural Science, Osaka Kyoiku University, Kashiwara 582-8582, Japan; ‡ Graduate School of Biological Sciences, Nara Institute of Science and Technology, Ikoma 630-0101, Japan

**Keywords:** *Brassica rapa* L, unilateral incompatibility, self-incompatibility, pollen-stigma recognition

## Abstract

Plants have evolved many systems to prevent undesirable fertilization. Among these, incompatibility is a well-organized system in which pollen germination or pollen tube growth is inhibited in pistils. We previously found that a novel one-way pollen–stigma incompatibility response [unilateral incompatibility (UI)] occurred between two self-incompatible *Brassica rapa* plants, a Turkish line, and a Japanese cultivated hybrid variety, “Osome.” Pollen from the Turkish line is rejected on the stigma of the Osome line, but the reverse cross is compatible; such a UI phenotype closely resembles self-incompatibility (SI). The pollen factor of this UI has been genetically explained by a single locus which is different from the *S*-locus. In this study, we performed further genetic analyses of this intraspecies UI and showed that the stigma factor was also controlled by a single locus, and we named the loci corresponding to the stigma and pollen factors of the intraspecies UI, stigmatic unilateral incompatibility (*SUI*), and pollen unilateral incompatibility (*PUI*) loci, respectively. Interestingly, segregation analyses of *SUI* and *PUI* indicated that they are closely linked to each other and behave as a single unit. To investigate the effect of an SI-related gene, *MLPK* in this UI, we produced segregation lines for *SUI* and *mlpk*. A distorted segregation ratio of SUI phenotype in an *mlpk* background indicated involvement of *MLPK* in SUI, suggesting the existence of an MLPK-dependent novel pollen–stigma recognition mechanism.

Flowering plants have evolved several pollen rejection mechanisms to prevent inappropriate pollination and fertilization. In such systems, pollen-pistil unilateral incompatibility (UI) is defined as a positive prefertilization barrier that results in pollen from one species being rejected on the stigma of another species, whereas a cross in the reciprocal combination is compatible (De Nettancourt 2001). In theory, this barrier prevents gene flow and is thus thought to underlie the maintenance of species identity. On the other hand, many plant species have self-incompatibility (SI) systems to prevent self-fertilization, which restricts inbreeding and generates genetic variation in a population. Recent studies of molecular mechanisms of pollen-pistil incompatibility have been focused mainly on the self-recognition system of SI (Iwano and Takayama 2012). Although molecular determinants for self-recognition from some plant species have been identified, including those of *Brassica* (Iwano and Takayama 2012), little information has been obtained for UI pollen-pistil recognition. Interspecies UI occurs between closely related species belonging to the same family, or cluster of families, hosting the same SI system. Incompatible pollination in UI is generally observed when an SI species is used as the stigma parent and a self-compatible (SC) species as the pollen parent. This relationship is called the “SI × SC rule,” although it cannot be applied in all cases (Lewis and Crowe 1958). In Solanaceae plants, which possess gametophytic SI, UI has also been reported in intraspecies crosses (Martin 1963).

In the SI system of Brassicaceae, self-pollen is rejected at the surface of stigmatic papilla cells after the discrimination of self- from non-self pollen. This recognition mechanism is genetically controlled by a single *S* locus, which contains two highly polymorphic genes, *SRK* (S-locus receptor kinase) and *SP11*/*SCR* (S locus protein 11 or S locus cysteine-rich protein) (Iwano and Takayama 2012). The pollen SI determinant SP11/SCR is a small cysteine-rich pollen coat protein which is expressed in sporophytic anther tapetum cells (Suzuki *et al.* 1999; Schopfer *et al.* 1999; Takayama *et al.* 2000b). This expression pattern governs sporophytic control of the *Brassica* SI system (Shiba *et al.* 2001; Tarutani *et al.* 2010). The stigma SI determinant SRK is a membrane-spanning serine/threonine receptor kinase (Stein *et al.* 1991; Takasaki *et al.* 2000). *S*-genotype-specific physical interaction between SP11/SCR, and a receptor domain of SRK is thought to trigger a signal cascade leading to the rejection of self-pollen on the stigma (Kachroo *et al.* 2001; Takayama *et al.* 2001; Shimosato *et al.* 2007). In the *Brassica* system, downstream cellular pathways and the target of SRK leading to self-pollen rejection are becoming better understood. One of these, the *M*-locus protein kinase (MLPK), which was isolated by positional cloning of the *M* locus of an SC *B. rapa* var. yellow sarson, is an essential positive regulator of the SI response (Murase *et al.* 2004). *MLPK* encodes a cytoplasmic serine/threonine protein kinase, which is expressed predominantly in the stigma, targets to the plasma membrane of stigmatic papilla cells, and colocalizes with SRK (Kakita *et al.* 2007).

It is known that the mechanism of SI is to some extent related to UI. In Solanaceae, several genetic studies have revealed that one of three factors that plays an important role in pistil-side UI mapped to near the *S*-locus region, although the other two factors were located in different loci (Bernacchi and Tanksley 1997). A transgenic experiment using the female SI determinant *S*-RNase (*S*-ribonuclease; female determinant of SI in Solanaceae and Rosaceae) clearly demonstrated that both *S* gene-dependent and *S* gene-independent mechanisms are involved in interspecies incompatibility in Solanaceae species (Murfett *et al.* 1996). Furthermore, discrete pollen-side UI factors in Solanaceae members have been mapped at or near the *S*-locus on chromosome 1, and another has been located on chromosome 6 (Chetelat and De Verna 1991). The factor mapped to chromosome 6 has been cloned and was found to carry a pollen-expressed Cullin1 gene with high similarity to petunia SI factors (Hua and Kao 2006; Li and Chetelat 2010). In the genus *Brassica*, Hiscock and Dickinson (1993) reported some phenotypic correlations between SI and interspecies UI. On the other hand, recent QTL analysis showed that the *S* and *M* loci are not involved in interspecies UI between *B. rapa* and *B. oleracea* (Udagawa *et al.* 2010). These multiple and redundant aspects of the UI phenomenon make interpretation of experimental results difficult. Thus, for an overall understanding of the UI mechanism, it will be necessary to dissect carefully defined UI systems on both the pollen and stigma sides.

We previously reported the novel intraspecies UI response that occurred between a Turkish line and a Japanese cultivated hybrid variety, Osome, in *Brassica rapa* (Takada *et al.* 2005). Pollen from the Turkish line was rejected on the stigma of some Osome-derived individuals, but crosses in the reverse combination were compatible. The physiological features of the UI closely resembled those of the SI responses in *Brassica*. Genetic analysis revealed that the factor controlling the pollen-side UI [pollen unilateral incompatibility (PUI)] is regulated by a single *PUI* locus, which is not linked to the *S* locus (Takada *et al.* 2005). Further genetic analysis of the stigma-side incompatibility [stigmatic unilateral incompatibility (SUI)] is required to isolate the *PUI* and *SUI* genes, which will lead to greater understanding of the novel pollen-stigma recognition mechanism of the intraspecies UI.

Herein, based on linkage analysis using F_2_ progeny between the *SUI*/*SUI* homozygote and the *sui*/*sui* homozygote, we concluded that SUI is controlled by a single dominant locus, which is not linked to the *S* locus. Segregation analysis for the *SUI* and *PUI* loci revealed that these two loci are closely linked to each other. In addition, to investigate the effect of *MLPK* in the SUI, we produced segregating progeny for *SUI* and *mlpk*. The biased segregation ratio of SUI phenotype in the *mlpk*/*mlpk* background suggested an MLPK-dependent incompatibility system operates in the SUI reaction.

## Materials and Methods

### Plant materials

Detailed genotypes of plant materials used in this study are shown in Table 1. Because we hypothesized that a positive interaction between female and male factors resulted in the UI reaction, the indicated genotypes of *SUI*, *sui*, *PUI*, and *pui* corresponded to the phenotype of SUI (incompatible), non-SUI (compatible), PUI (incompatible), and non-PUI (compatible), respectively. It has not yet been determined whether PUI is regulated sporophytically or gametophytically (see *Discussion*). For the SUI homozygous line, *S^60^*-9 and *S^52^*-12 were self-pollinated progeny of *B. rapa* cv. Osome (Takasaki *et al.* 1999; Takada *et al.* 2005). The original *B. rapa* cv. Osome line is heterozygous for *S^60^* and *S^52^ S*-haplotype. *S^60^* and *S^52^* homozygous lines were selected from self-crossed progeny of *B. rapa* cv. Osome by using allele-specific polymerase chain reaction (PCR) and test pollination. In our previous study, *S^60^*-9 and *S^52^*-12 were characterized as possessing the SUI phenotype (Takada *et al.* 2005). We checked the SUI phenotype of the self-crossed progeny of *S^60^*-9 and *S^52^*-12, and established that these are both SUI homozygous lines. The non-UI line *S^9^* was derived from Oguni, Japan (Nou *et al.* 1993; Hatakeyama *et al.* 1998). For the *S^9^*UI line, an *SUI*/*SUI*, *S^9^*/*S^9^* homozygous plant was selected from the F_2_ segregation line of SUI line (*S^60^*-9) and non-SUI line (*S^9^*) (see Supporting Information, Figure S1). *PUI* homozygous lines [*S^24^*t and *S^40^*t (where t stands for Turkey)] were obtained from self-pollinated progeny of the Turkish variety with *S^24^* and *S^40^* background, respectively (Nou *et al.* 1993; Hatakeyama *et al.* 1998; Takada *et al.* 2005). The *mlpk* mutant *S^8^*mm was reported by Murase *et al.* (2004).

**Table 1:** Plant materials and their expected genotypes

Line	Name	Expected Genotype	Study(ies)
SUI	*S^60^*-9	*SUI*/*SUI*, *pui*/*pui*, *MLPK*/*MLPK*, *S^60^/S^60^*	Takada *et al.* 2005
	*S^52^*-12	*SUI*/*SUI*, *pui*/*pui*, *MLPK*/*MLPK*, *S^52^/S^52^*	Takada *et al.* 2005
	*S^9^*UI	*SUI*/*SUI*, *pui*/*pui*, *MLPK*/*MLPK*, *S^9^/S^9^*	This report
PUI	*S^24^*t	*sui*/*sui*, *PUI*/*PUI*, *MLPK*/*MLPK*, *S^24^/S^24^*	Takada *et al.* 2005; Hatakeyama *et al.* 1998
	*S^40^*t	*sui*/*sui*, *PUI*/*PUI*, *MLPK*/*MLPK*, *S^40^/S^40^*	Takada *et al.* 2005; Hatakeyama *et al.* 1998
non-UI	*S^9^*	*sui/sui*, *pui/pui*, *MLPK/MLPK*, *S^9^/S^9^*	Takada *et al.* 2005; Hatakeyama *et al.* 1998
*mlpk* mutant	*S^8^*mm	*sui*/*sui*, *pui*/*pui*, *mlpk*/*mlpk*, *S^8^/S^8^*	Murase *et al.* 2004

### Production of segregating progeny and test pollinations

**Figure 1:**
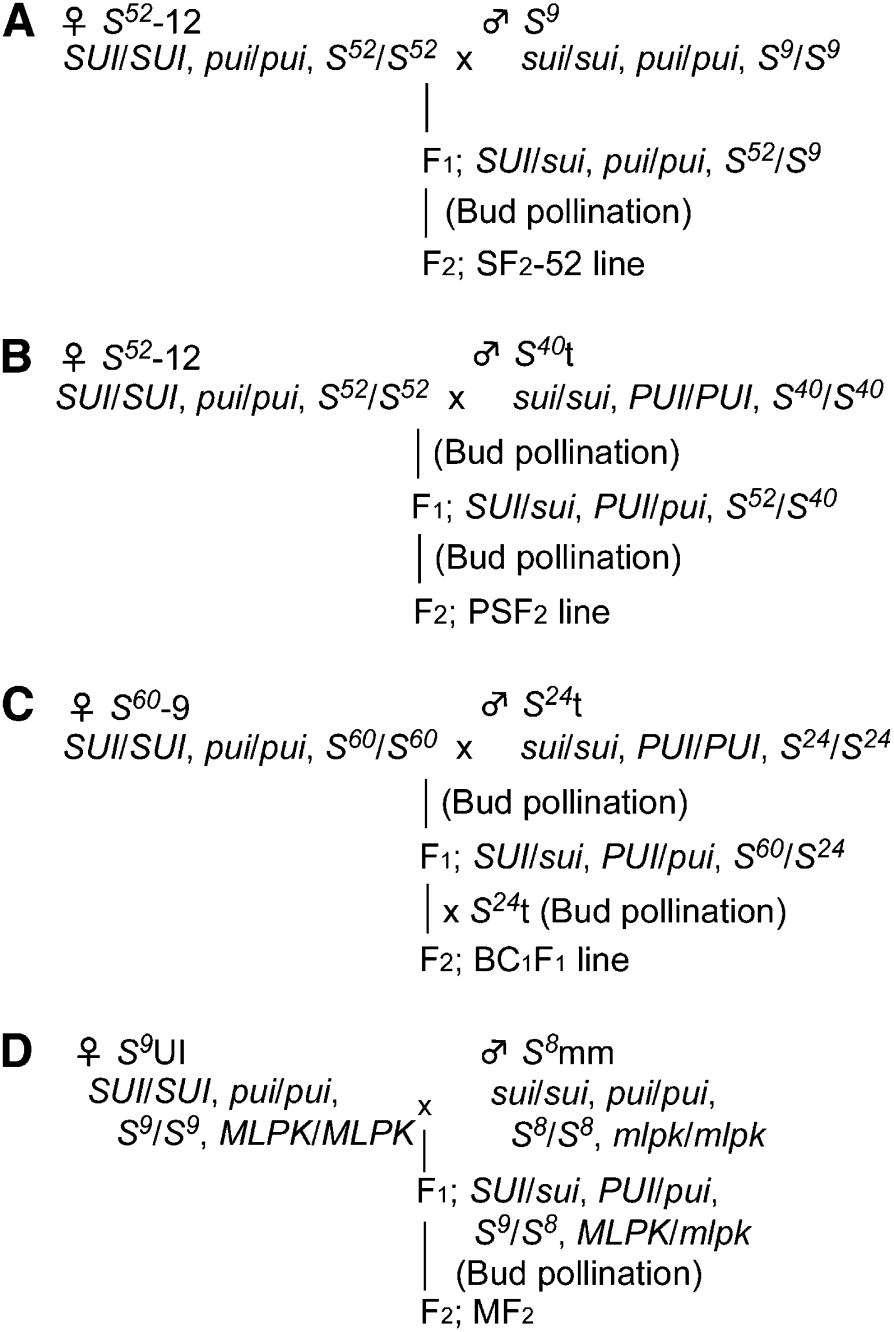
Crossing scheme used to develop each segregation line.

The crossing scheme is represented in Figure 1. For the production of *SUI* segregating progeny termed SF_2_-52 (stigma side F_2_ line with *S^52^* genotype) and SF_2_-60 (stigma side F_2_ line with *S^60^* genotype), *S^52^*-12 or *S^60^*-9 was crossed with *S^9^* to produce F_1_ plants, which were then self-crossed by bud pollination to produce F_2_ seeds (Figure 1A; Figure S1). *SUI* and *PUI* segregating progeny, termed the pollen-stigma side F_2_ (PSF_2_) line, was produced by self-bud pollination of an F_1_ plant, which was produced by bud pollination of *S^52^*-12 and *S^40^*t (Figure 1B). For production of the backcross C_1_F_1_ (backcross_1_F_1_) line, we performed bud pollination of *S^60^*-9 stigma with *S^24^*t pollen. Thereafter, the F_1_ plant was bud-pollinated with *S^24^*t pollen (Figure 1C). In the case of *SUI* and *MLPK* (MF_2_ line), *S^9^*UI and *S^8^*mm were used as parental plants. Subsequently, we performed bud pollination of the F_1_ plant (Figure 1D). SUI, PUI phenotypes, and *S*-genotypes of all plants used in this study were determined by test pollination as described in Takada *et al.* (2005). Flower buds were numbered from the lowest bud in an inflorescence, as described in Gonai and Hinata (1971). Because the average number of flowers opening per day is three, the flower buds at stages 1′, 2′, and 3′ are expected to bloom on the next day, and stigmas of these three buds have the potential to exhibit a normal SI reaction (Gonai and Hinata1971). Nonpollinated flower buds were cut at the peduncle and, after pollination, stood on 1% solid agar for approximately 24 hr under room conditions. Thereafter, pistils of the pollinated flowers were softened in 1 N NaOH for 1 hr at 60°C and stained with basic aniline blue (0.1M K_3_PO_4_, 0.1% aniline blue). Samples were mounted in 50% glycerol (fluorescence microscope grade) on slides and observed by UV fluorescence microscopy (Kho and Bear 1968). At least three flowers were used in each cross combination, and observations were generally replicated at least three times for each cross combination on different dates. For determination of the SI phenotype, *S*-homozygous plants, *S^9^*, *S^24^*, *S^40^*, *S^52^*, and *S^60^*, were used. To determine the UI phenotype, *PUI* homozygous (*S^24^*t or *S^40^*t) and *SUI* homozygous (*S^52^*-12 or *S^60^*-9) plants were used in the test pollination (Takada *et al.* 2005).

### Seed production by test crossing using mature stigmas

To avoid self-pollination in the bud, 1 day before opening the stamens were removed from three flowers corresponding to stages −1′, −2′, and −3′. The inflorescence was then covered with a paper bag for 24 hr. After pollination, we excised all flowers in the inflorescence with the exception of the three flowers and re-covered the inflorescence with a paper bag for approximately 3 weeks. For a comparison of the incompatibility strength, we measured the lengths of siliques and counted the number of seeds.

### Determination of the *S* genotype and *MLPK* genotype

The *S*-genotype of each plant was determined by allele-specific PCR. Total DNA was extracted from young leaf tissue of *B*. *rapa* by the procedure described by Murray and Thompson (1980). PCR was performed using r*Taq* DNA polymerase (Takara BIO, Shiga, Japan) for 30 cycles of denaturation at 94°C for 30 sec, annealing at 56°C for 20 sec, and extension at 72°C for 30 sec. Genomic DNA of each plant was amplified using *S*-genotype-specific *SP11* primers. For genotyping of *MLPK*, the functional *MLPK* allele was specifically amplified by using the PCR primers wtMLPK-F and wtmMLPK-R, and the mutated *mlpk* allele was specifically amplified by the mMLPK-F and wtmMLPK-R primers. PCR primers used in this study are listed in Table S1. The PCR product was subjected to electrophoresis on 1% agarose gel. Vacuolar H^+^-ATPase (V-ATPase) of *B. rapa* (Takasaki *et al.* 2000) was amplified as a positive control.

### Statistical analysis

Statistical analyses were performed using Statistica version 6.1 software (StatSoft Inc., Tulsa, OK). Tukey’s honestly significant difference test was used to determine significant differences. Pearson’s correlation coefficient (*r*) values were calculated between silique length and seed number.

For the segregation analysis, a standard χ^2^ goodness-of-fit test was performed to determine if phenotype or genotype distribution deviated from expected ratios.

## Results

### Seed production of UI combination crosses

**Figure 2:**
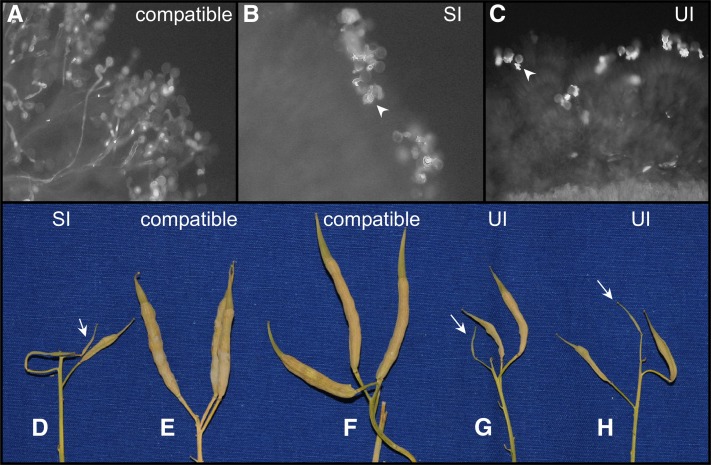
Representative results of test pollinations and seed production. Photographs were obtained by UV fluorescence microscopy (A–C). (A) Cross-pollination of an *S^24^*t stigma with *S^52^*-12 pollen. (B) Self-pollination of *S^52^*-12. (C) Cross-pollination of an *S^52^*-12 stigma with *S^24^*t pollen. The pollen germinated, but penetration of the pollen tube was inhibited (arrowheads). Silique length of each test cross (D–H). (D) *S^60^*-9 self-cross; (E) *S^60^*-9 stigma × *S^9^* pollen; (F) *S^60^*-9 stigma × *PUI*/*pui* heterozygous pollen; (G) *S^60^*-9 stigma × *S^24^*t; (H) *S^52^*-12 stigma × *S^40^*t. Short siliques were observed in SI and UI crosses (arrows).

Our previous experiments showed that the UI phenotype in *B. rapa* closely resembles the SI phenotype on the stigmatic papillae cells (Takada *et al.* 2005). In contrast to compatible pollination, the incompatible pollen cannot germinate and/or cannot penetrate stigmatic papillae cells in the SI and UI reaction (Figure 2, A, B, and C). Moreover, the UI pollen rejection can be overcome by bud pollination, as in the case of SI. In this study, to test the effect of UI on seed production, we performed test crosses between the SUI stigma and the PUI pollen. The silique length and number of seeds were measured in each test cross. A strong correlation (*r* = 0.86) was detected between silique length and number of seeds (Table 2; File S1). In the SI cross (*S^60^*-9 self), only short siliques were obtained, and the average seed number was 0.9 (Figure 2D; Table 2), while fully developed long siliques were observed in a compatible combination (cross-pollination: *S^24^*t stigma × *S^52^*-12 pollen) (Figure 2E; Table 2). The UI cross (*S^52^*-12 stigma × *S^24^*t pollen) resulted in production of short siliques (Figure 2, G and H, and Table 2), which could not be distinguished from those of the SI combination cross. There were no significant differences between SI and UI in silique length or seed number (Table 2). These results indicate that UI shows the same strength of incompatibility as SI in terms of seed production. When we crossed pollen from *PUI/pui* heterozygous plants (S*^29^*/*S^40^*t, *PUI*/*pui*) (Takada *et al.* 2005) with mature *SUI* stigmas, full seed sets was observed, as occurred in the SC combination (Figure 2F). This was consistent with our previous report showing compatible behavior of *PUI*/*pui* heterozygous pollen on the *SUI* stigma (Takada *et al.* 2005).

**Table 2:** Seed Production by UI and SI Phenotypes

Cross	Silique Length (cm)	Mean Number of Seeds (±SD)	*n*
Compatible cross	5.5 ± 0.5*^a^*	15.3 ± 2.7*^c^*	6
Self-incompatible cross	2.8 ± 0.9*^b^*	0.9 ± 0.9*^d^*	9
Unilateral incompatible cross	3.0 ± 1.1*^b^*	1.2 ± 1.5*^d^*	11

Seed production phenotypes were measured by silique length and mean number of seeds (± SD). Values followed by the same letter are not significantly different at the 5% level, as determined by Tukey’s multiple comparison test.

### Genetic segregation analysis of the SUI factor

**Figure 3:**
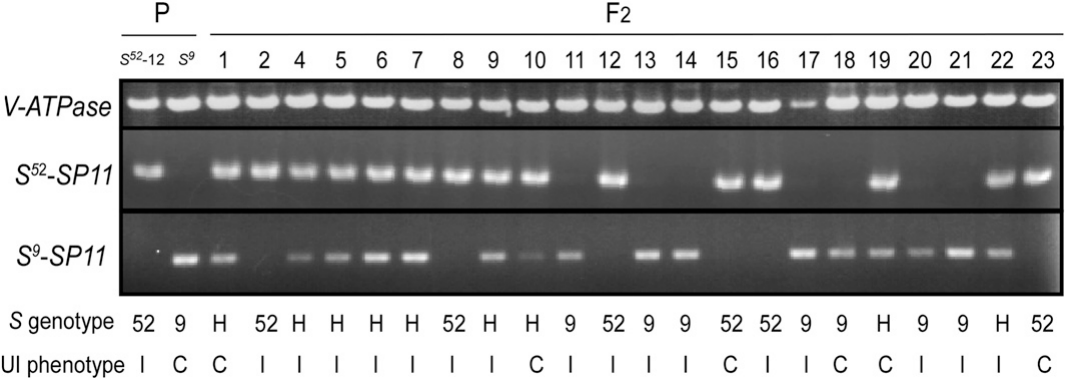
The SUI is regulated by an *S*-locus-independent single *SUI* locus. Genomic DNA isolated from parental (P) plants homozygous for either the *S^52^*-12 or *S^9^* and 23 SF_2_-52 progeny plants (F_2_), and their *SP11* were amplified using their *S*-genotype-specific primer. The SUI phenotype of the stigma of each plant to *S^24^*t pollen was determined by pollination tests. The *S* genotype and SUI phenotype are shown below each lane: 52, *S^52^*-homozygote; 9, *S^9^*-homozygote; H, *S^52^*/*S^9^*-heterozygote; C, compatible with *S^24^*t pollen (non-UI); I, incompatible with *S^24^*t (UI). *V-ATPase* gene was amplified as a positive control.

The UI reaction at the pollen side is regulated by a single *PUI* locus, which is not linked to the *S* locus (Takada *et al.* 2005). Furthermore, in our previous report, the stigma UI phenotype was shown to occur in both the *S^60^*/*S^60^* and *S^52^*/*S^52^* backgrounds (Takada *et al.* 2005), suggesting the possible independent segregation of *SUI* and *S* genotypes. In order to determine the genetic control of SUI and confirm nonlinkage to the *S* locus, we performed a segregation analysis using the F_2_ generation between the SUI *S^52^*-12 (*SUI*/*SUI*, *pui*/*pui*, *S^52^*/*S^52^*) and the non-SUI *S^9^* (*sui*/*sui*, *pui*/*pui*, *S^9^*/*S^9^*) lines (Figure 1A). The SF_2_-52 progeny was produced by self-bud pollination of the F_1_ heterozygous plant (*SUI*/*sui*, *pui*/*pui*, *S^9^*/*S^52^*). Stigma UI phenotypes of SF_2_-52 segregating progeny and F_1_ plants were determined by test cross-pollination using *PUI*/*PUI* homozygous plants (*S^24^*t) as the pollen donor. The stigma phenotype of all the 11 F_1_ plants was incompatible with the PUI pollen, indicating that the SUI trait was dominant over normal cross compatibility. The stigma phenotype of the SF_2_-52 progeny clearly segregated as unilateral incompatibility (SUI) or cross compatibility (non-SUI) (Figure 3; File S2). According to χ^2^ test results, the SUI:non-SUI segregation ratio of 146:46 fitted to the single locus segregation ratio of 3:1 (χ^2^ = 0.11, *P* > 0.05, *df* = 1). In this progeny, the *S*-genotype determined by the allele-specific *SP11* amplification segregated normally. The SUI phenotype and *S* genotype in the SF_2_-52 individuals segregated independently (Figure 3), indicating that the *SUI* locus is not linked to the *S* locus, which is consistent with our previous crossing experiments (Takada *et al.* 2005). Furthermore, the *S*-locus-independent segregation of the SUI phenotype was confirmed by using *S^60^ S*-genotype background (Figure S1, File S3). Thus, the stigma UI phenotype is genetically controlled by a single dominant *SUI* locus, which is not linked to the *S* locus.

### Linkage analysis of the *SUI* and *PUI* loci

**Figure 4:**
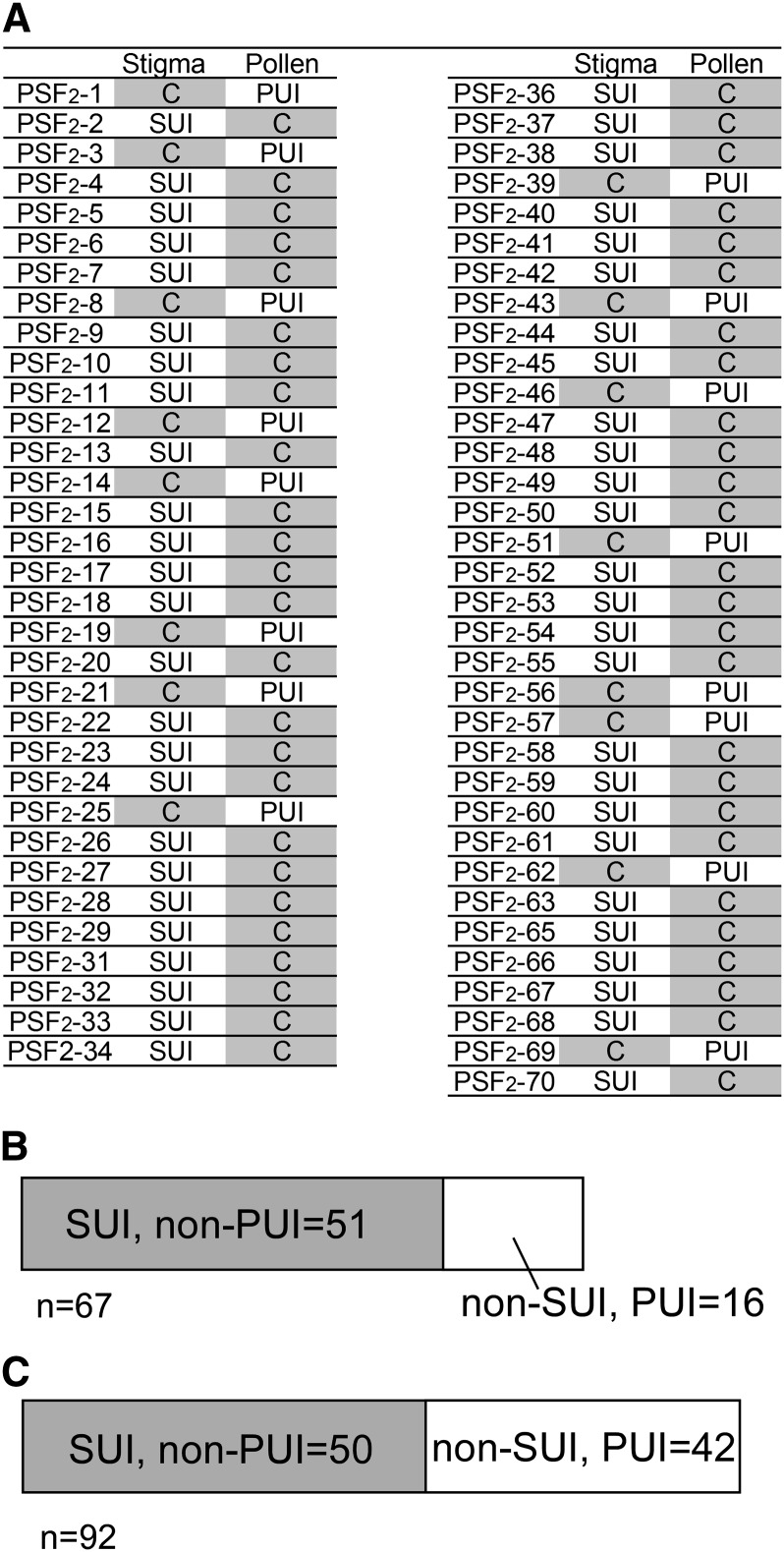
(A–C) *SUI* and *PUI* loci are closely linked to each other. The PSF_2_ segregation line was produced by a cross between *S^52^*-12 and *S^40^*t (as shown in Fig. 1). (A) The UI phenotypes of the stigma side and pollen side were determined by using *S^60^*-9 as the stigma tester plant and *S^24^*t as the pollen tester plant. SUI, Stigmatic UI; PUI, pollen UI; C (shaded boxes), compatible. (B) Segregation ratio of the PSF_2_ line. The SUI, PUI or non-SUI, non-PUI progeny was not found. (C) Segregation ratio of the BC_1_F_1_ line.

Our segregation analysis clearly showed that both the female and male factors of *Brassica* intraspecies UI are regulated by a single locus. This reminded us of the SI recognition mechanism in which *SRK* and *SP11* (genes for female and male SI factors, respectively) are tightly linked on the *S*-locus region and are inherited as a single segregation unit because recombination within this region is highly repressed. In order to examine the relationship between the *SUI* and *PUI* loci, we further performed linkage analysis using a segregating population derived from a plant heterozygous for both *SUI* and *PUI* (*SUI*/*sui* and *PUI*/*pui*). The F_1_ heterozygous plant was produced by self-bud pollination between *S^52^*-12 (*SUI*/*SUI*, *pui*/*pui*, *S^52^*/*S^52^*) and *S^40^*t (*sui*/*sui*, *PUI*/*PUI*, *S^40^*/*S^40^*), and the F_2_ progeny (PSF_2_) was produced by self-pollination of the F_1_ heterozygous plant (*SUI*/*sui*, *PUI*/*pui*, *S^52^*/*S^40) (^*Figure 1B). Stigma- and pollen-UI phenotypes of each plant were determined by test pollination using *S^24^*t (*sui*/*sui*, *PUI*/*PUI*, *S^24^*/*S^24^*) as a pollen donor and *S^60^*-9 (*SUI*/*SUI*, *pui*/*pui*, *S^60^*/*S^60^*) as a stigma recipient. These tester plants were chosen in order to discriminate between UI and SI responses. In the PSF_2_ progeny, all of the PUI individuals showed a non-SUI phenotype, and all of the pollen from SUI individuals was compatible on SUI tester stigma, indicating the tight linkage of the *SUI* and *PUI* loci (Figure 4A). There were no plants showing both SUI and PUI phenotypes in the 67 PSF_2_ plants. The phenotypic SUI/non-PUI:non-SUI/PUI segregation ratio was 51:16 (Figure 4B), clearly matching to single-locus inheritance (3:1; χ^2^ = 0.048; *P* > 0.05; *df* = 1). For further confirmation of this linkage, we used another segregating progeny (BC_1_F_1_), which was produced by bud pollination using the heterozygous F_1_ (*SUI*/*sui*, *PUI*/*pui*, *S^60^*/*S^24^*) stigma and *PUI* homozygous (*sui*/*sui*, *PUI*/*PUI*, *S^24^*/*S^24^*) pollen (Figure 1C). Results of test crosses of 92 BC_1_F_1_ individuals revealed that the phenotypic segregation ratio of SUI/non-PUI and non-SUI/PUI was 50:42 (1:1 by χ^2^ = 0.69; *P* > 0.05; *df* = 1), and complete linkage was observed with the *SUI* allele and *pui* allele from *S^60^*-9, and *sui* allele and *PUI* allele from *S^24^*t (Figure 4C; File S4). A plant possessing both SUI stigma and PUI pollen, or non-SUI stigma and non-PUI pollen, was not present in the 67 F_2_ progeny and 92 BC_1_F_1_. Therefore, recombination between *SUI* and *PUI* was not observed. These results clearly indicate a strong linkage between the *SUI* and *PUI* loci.

### Effect of *MLPK* on SUI pollen rejection

Due to the phenotypic resemblance between SI and this intraspecies UI, it is possible that the SI and UI systems use common cellular targets to reject the pollen. To investigate the effect of *MLPK*, whose product is one of the important targets of the SI system (Murase *et al.* 2004), on this UI we produced segregating progeny for *SUI* and *MLPK*. F_1_ plants heterozygous for the *SUI* locus and the *MLPK* locus were produced by crossing an *S^9^*UI plant (*SUI*/*SUI*, *pui*/*pui*, *S^9^*/*S^9^*, *MLPK*/*MLPK*) and an *S^8^*mm plant (*sui*/*sui*, *pui*/*pui*, *S^8^*/*S^8^*, *mlpk*/*mlpk*) (Figure 1D). The stigma phenotype of the F_1_ heterozygous plant (*SUI*/*sui*, *pui*/*pui*, *S^8^*/*S^9^*, *MLPK*/*mlpk*) was incompatible with the PUI tester pollen (*S^40^*t), in the same manner as that expected with UI. In the MF_2_ segregation line, *S*-genotypes segregated as expected: *S^8^*/*S^8^*:*S^8^*/*S^9^*:*S^9^*/*S^9^* = 22:34:16 (1:2:1; χ^2^ = 1.21; *P* > 0.05; *df* = 2) (Table 3; File S5). The overall segregation of SUI phenotype in the MF_2_ line did not fit a 3:1 ratio (3:1; χ^2^ = 7.41; *P* < 0.01; *df* = 1) but was consistent with a 9:7 ratio (9:7; χ^2^ = 0.689; *P* > 0.05; *df* = 1). This suggests that functional *SUI* and *MLPK* are both essential for the UI pollen rejection. The UI phenotypes of plants possessing a functional *MLPK* allele (*MLPK*/*MLPK* and *MLPK*/*mlpk*) segregated in a SUI:non-SUI 3:1 ratio, indicating that the *SUI* locus is located at a different chromosomal position from *MLPK* (Table 3). In the case of the nonfunctional *mlpk* background (*mlpk*/*mlpk*), 12 of 15 plants showed a compatible phenotype to PUI tester pollen, although the other 3 plants remained UI (Table 3). Here the altered SUI observed in most of the plants was associated with *mlpk*/*mlpk*-dependent disruption of the incompatible reaction. Thus, it is suggested that an MLPK-dependent incompatibility system may have an important role in the rejection of PUI pollen on SUI stigmas in *B. rapa*. Moreover, it is noteworthy that 3 plants showed SUI in the *mlpk*/*mlpk* background, indicating the possible existence of an additional signal cascade, which is MLPK-independent, in this UI system (Table 3). To confirm the interaction between *MLPK* and UI pollen rejection, we further selected 13 plants possessing *mlpk*/*mlpk* homozygous background in the MF_2_ line and checked their SUI phenotype. All plants were compatible with PUI tester pollen, apart from one that still showed SUI pollen rejection (File S6).

**Table 3:** Segregation of SUI in *mlpk*/*mlpk* SC Mutant

*M* Genotype	*S* Genotype	SUI Phenotype		Expected Ratio	χ^2^ Results
		SUI	Non-SUI		
*MLPK*/*MLPK*	*S^9^*/*S^9^*	2	2		
*MLPK*/*MLPK*	*S^9^*/*S^8^*	7	3		
*MLPK*/*MLPK*	*S^8^*/*S^8^*	7	1		
		16	6	3:1	0.06*
*MLPK*/*mlpk*	*S^9^*/*S^9^*	4	5		
*MLPK*/*mlpk*	*S^9^*/*S^8^*	14	1		
*MLPK*/*mlpk*	*S^8^*/*S^8^*	7	4		
		25	10	3:1	0.23*
*mlpk*/*mlpk*	*S^9^*/*S^9^*	1	2		
*mlpk*/*mlpk*	*S^9^*/*S^8^*	2	7		
*mlpk*/*mlpk*	*S^8^*/*S^8^*	0	3		
		3	12	3:1	24.19**

* *P* > 0.05; **P < 0.001.

## Discussion

In the present genetic study, we have characterized the novel stigma-pollen interaction between SUI and PUI factors in *B. rapa*, which is similar to SI in the following points: (1) UI is regulated by a single locus with the tightly linked stigma and pollen factor genes *SUI* and *PUI*. (2)The incompatibility phenotype of UI on the stigma is quite similar to that of SI, and bud pollination can be used to overcome UI. (3) MLPK may be involved in the SUI reaction. Although this UI is similar to SI, the *SUI*/*PUI* locus is not linked to the *S* locus, and there is no evidence for multiple alleles. It would be interesting to discuss the evolution of this UI, if the *SUI* and *PUI* genes could be identified.

In our series of genetic analyses, the SUI phenotype was found to be genetically controlled by a single dominant locus. The recognition and rejection of the *PUI*/*PUI* pollen specifically occurred on the stigma surface by action of the SUI determinant. In the *SUI*/*SUI* homozygous or *SUI*/*sui* heterozygous plant, the *SUI* gene would be expressed in stigmatic papillae cells, as in the case of *SRK* in the SI system. Therefore, SRK-like receptor kinase is a possible candidate for the SUI factor. In *Arabidopsis*, *S*-domain receptor kinases fall into three classes with more than 40 members (Shiu and Bleecker 2003), and the functions of this family of kinases remain largely unknown. In *Brassica*, at least two *SRK*-like genes that are not linked to the *S*-locus have been reported, and one of these was found to be expressed predominantly in stigma (Kai *et al.* 2001). It is possible that the *SUI* gene is one of the uncharacterized members of such a *Brassica S* multigene family.

The pollen determinant is regulated by a single *PUI* locus, and it appears to be a recessive phenotype in the heterozygous *PUI*/*pui* plant (Takada *et al.* 2005). However, it should be noted that there is the possibility of a pseudorecessive phenotype; if *PUI* is expressed gametophytically in pollen grains, the heterozygote (*PUI*/*pui*) will produce pollen grains having *PUI* or *pui*, and *PUI* pollen will be inhibited, whereas *pui* pollen will be compatible. In this case, half of the pollen grains can germinate, and their pollen tubes can penetrate the stigma, which would be judged compatible in the test pollination assay. Thus, if *PUI* is gametophytically regulated, its behavior will be similar to that of the recessive in the heterozygote. To date, it has been unclear whether PUI is regulated sporophytically or gametophytically. If the UI recognition and rejection system is the result of a positive interaction between stigma and a pollen-producing product, the PUI could be controlled gametophytically. As the candidate for the PUI factor in *B. rapa* expressed in pollen grains, several signaling molecules have been reported. Over 10 different small basic pollen coat proteins (PCPs) have been identified in *Brassica* pollen coat (Doughty *et al.* 1998; Takayama *et al.* 2000a). One of these PCPs, PCP-class A, 1 (PCP-A1) was reported to be expressed gametophytically in pollen grains and had the ability to bind to *S*-locus glycoprotein (SLG) and possibly SRK (Doughty *et al.* 1998, 2000). Another member of the PCPs, *S* locus-related glycoproteins 1-binding protein (SLR1-BP) has also been reported to have an important role in pollen adhesion to papillae cells and can physically interact with the SLG-like protein SLR1 (Takayama *et al.* 2000a). In view of these reports, it would interesting to determine the expression and genetic control of the *PUI*, as this may be different from *SP11*, the pollen SI factor, which is sporophytically controlled.

Interestingly, analysis of linkage between *SUI* and *PUI* clearly indicated that these two loci cosegregate and act as a one inheritance unit. There are at least three types of genomic structure in the locus: (1) non-UI in both the stigma and pollen (Japanese lines and cv. Osome non-SUI type; *sui*/*pui*); (2) stigmatic SUI and pollen non-PUI (cv. Osome SUI type; *SUI*/*pui*); and (3) stigmatic non-SUI and pollen PUI (Turkish lines; *sui*/*PUI*). We have not been able to find a plant or line that has both active SUI and PUI.

To the best of our knowledge, this is the first report of novel pollen-stigma interacting factors, which are located in the same or closely linked loci, other than the SI system. Segregation analysis of *SUI* and *MLPK* clearly indicated that SI and UI share the same signal cascade in stigmatic papilla cells’ ability to reject pollen. These findings suggest a possible ligand receptor recognition system similar to that of SI in this UI. In addition, it is reported that in the genome of *A. thaliana*, there are some regions where *SRK*-like genes and several SP11-like genes, duplicated in tandem, are located in closely linked states (Zhang *et al.* 2011). In our possible hypothesis of the intraspecies UI in *B. rapa*, SRK-like receptor kinase expressed in the SUI stigma interacts with an SP11-like peptide expressed gametophytically in PUI pollen, triggering the MLPK-involved signaling pathway to induce a rapid incompatible reaction in the papilla cells.

In the segregation analysis of *SUI* and *MLPK*, unexpectedly, we found some plants possessing *mlpk*/*mlpk* homozygous background and still showing SUI to the PUI tester pollen. This suggests that a minor *MLPK*-independent pollen rejection system is also involved in intraspecies UI in *B. rapa*. A simple explanation of this system would be that a locus other than *MLPK* plays a role in UI. Because the plants that we used in this work are not inbred lines or doubled haploids, they contain some degree of heterozygosity. These genetic variations might be involved in an *MLPK*-independent UI system. Udagawa *et al.* (2010) reported that functional *MLPK* is not needed for the interspecies UI between *B. rapa* and *B. oleracea*. Although both inter- and intraspecies UI in *Brassica* represent a one-way incompatibility response in stigmatic papilla cells, it is considered possible that intraspecies UI in this study might be regulated by a partially different pollen-stigma incompatibility mechanism from the interspecies UI.

In this study, we also characterized a seed production phenotype of the intraspecies UI in *B. rapa*, in which the strength of UI could not be distinguished from that of SI. If SUI and PUI lines are grown in the same field, their hybrid seeds should not be produced on the SUI line. The UI pollen rejection system described here would potentially function as a reproductive barrier between the *PUI*-possessing Turkish line and *SUI*-possessing Japanese commercial line, although it is not a complete barrier.

The SI system is widely used for production of *Brassica* hybrid seeds, and the strength of SI is an important phenotype for efficient breeding of F_1_ hybrid crops. Several genetic studies of the variation in strength of the SI response have been reported (Hatakeyama *et al.* 2010; Isokawa *et al.* 2010), suggesting that there are several genetic factors regulating SI strength in *Brassica*. From the viewpoint of incompatibility strength, UI described here has a strong incompatible response, the same as in the rigid SI phenotype. Potentially, by using this *UI* locus for breeding of *Brassica* crops, alternative efficient systems for F_1_ breeding could be available in the future. Identification and characterization of the molecular determinants of this *Brassica* intraspecies UI will contribute to our understanding of the molecular mechanisms of the pollen-stigma interaction and incompatibility.

## Supplementary Material

Supporting Information
