# Supplementary material for: Involvement of MLPK Pathway in Intraspecies Unilateral Incompatibility Regulated by a Single Locus With Stigma and Pollen Factors
Source: G3 (Bethesda). 2013 Apr 1;3(4):719–26. doi: 10.1534/g3.113.005892 (PMC3618358; doi:10.1534/g3.113.005892)
Supplement: Supporting Information [file supp_3_4_719__index.html]

Involvement of MLPK Pathway in Intraspecies Unilateral Incompatibility Regulated by a Single Locus With Stigma and Pollen Factors — Supporting Information 

# Involvement of MLPK Pathway in Intraspecies Unilateral Incompatibility Regulated by a Single Locus With Stigma and Pollen Factors

## Supporting Information for Takada *et al.*, 2013

**Files in this Data Supplement:**

- Supporting Information - Figure S1, Files S1-S6, and Table S1 (PDF, 211 KB)
- Figure S1 - Segregation analysis of SF2-60 (PDF, 122 KB)
- Table S1 - Primers (PDF, 138 KB)
- File S1 - The silique length and the number of seeds in each test cross (.xls, 14 KB)
- File S2 - SUI phenotypes and S genotypes of SF2-52 segregation lines to S24t or S40t PUI pollen (.xls, 20 KB)
- File S3 - SUI phenotypes and S genotypes of SF2-60 segregation lines to S24t or S40t PUI pollen (.xls, 14 KB)
- File S4 - SUI phenotypes of (S60-9xS24t) x S24t BC1F1 lines to S40t PUI pollen (.xls, 15 KB)
- File S5 - S-genotype, MLPK genotype and SUI phenotype of MF2 lines (.xls, 17 KB)
- File S6 - SUI phenotypes and S-genotypes of selected mlpk/mlpk plants from MF2 lines (.xls, 15 KB)
