# Supplementary material for: Involvement of MLPK Pathway in Intraspecies Unilateral Incompatibility Regulated by a Single Locus With Stigma and Pollen Factors
Source: G3 (Bethesda). 2013 Apr 1;3(4):719–26. doi: 10.1534/g3.113.005892 (PMC3618358; doi:10.1534/g3.113.005892)
Supplement: Supporting Information [file supp_g3.113.005892_005892SI.pdf]

G3·Genes | Genomes | Genetics

Involvement of MLPK pathway in intraspecies unilateral incompatibility regulated by a single locus with stigma and pollen factors.

Yoshinobu Takada*, Takahiro Sato*, Go Suzuki§, Hiroshi Shiba†[1], Seiji Takayama† and Masao Watanabe*[2]

*Graduate School of Life Sciences, Tohoku University, Sendai 980-8577, Japan,

§Division of Natural Science, Osaka Kyoiku University, Kashiwara 582-8582, Japan,

†Graduate School of Biological Sciences, Nara Institute of Science and Technology, Ikoma 630-0101, Japan

[1]Present address: Faculty of Science, Ibaraki University, Mito 310-8512, Japan.

[2]Corresponding author: Masao Watanabe

Laboratory of Plant Reproductive Genetics, Graduate School of Life Sciences, Tohoku University, 2-1-1, Katahira,

Aoba-ku, Sendai 980-8577, Japan.

E-mail: nabe@ige.tohoku.ac.jp

DOI: 10.1534/g3.113.005892

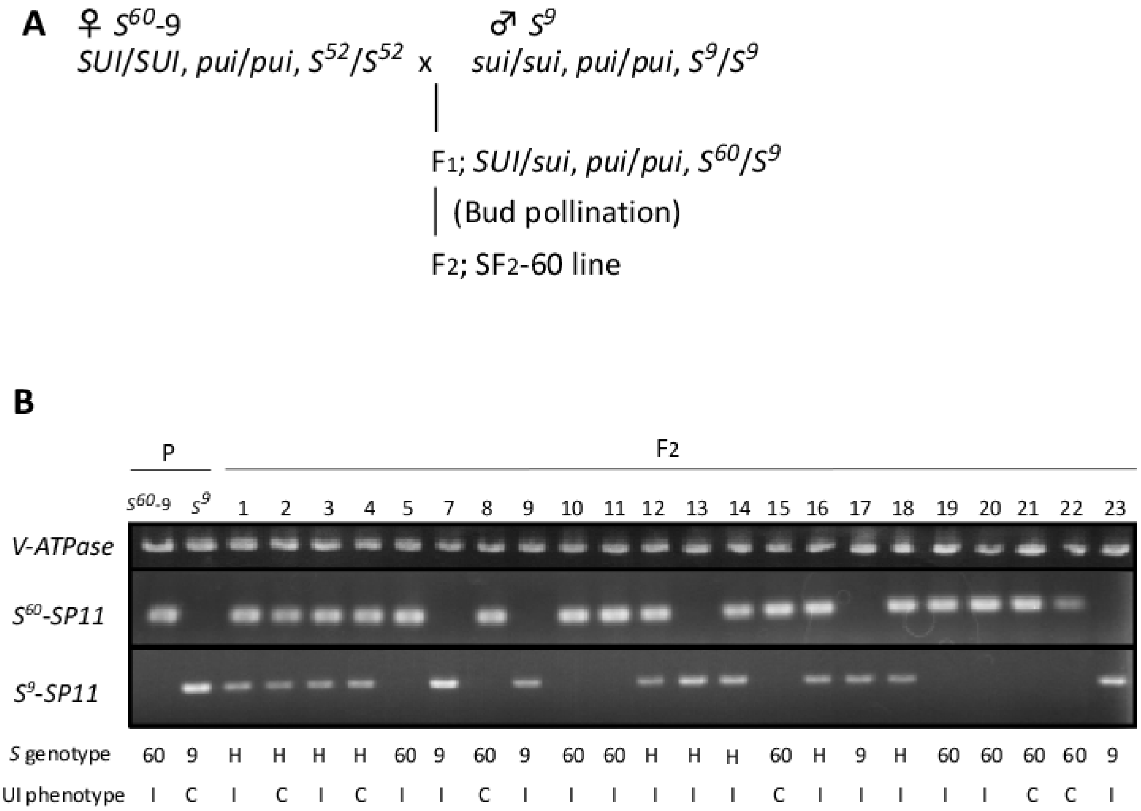

Figure S1 Segregation analysis of SF_2-60.

(A) Crossing scheme used to develop the SF_2-60 segregation line. (B) Genomic DNA isolated from parental (P) plants homozygous for either the S^{60}-9 or S^{9}, and SF_2-60 progeny plants (F_2) and their *SP11* were amplified using their *S*-genotype specific primer. The SUI phenotype of the stigma of each plant to S^{40}t pollen was determined by pollination tests. The *S* genotype and UI phenotype are shown below each lane: 60, S^{60}-homozygote; 9, S^{9}-homozygote; H, S^{60}/S^{9}-heterozygote; C, compatible to S^{40}t pollen (non-UI); I, incompatible to S^{24}t (UI). *V-ATPase* gene was amplified as a positive control.

Files S1-S6

Available for download at http://www.g3journal.org/lookup/suppl/doi:10.1534/g3.113.005892/-/DC1

File S1 The silique length and the number of seeds in each test cross.

File S2 SUI phenotypes and S genotypes of SF2-52 segregation lines to S24t or S40t PUI pollen.

File S3 SUI phenotypes and S genotypes of SF2-60 segregation lines to S24t or S40t PUI pollen.

File S4 SUI phenotypes of (S60-9xS24t) x S24t BC1F1 lines to S40t PUI pollen.

File S5 S-genotype, MLPK genotype and SUI phenotype of MF2 lines.

File S6 SUI phenotypes and S-genotypes of selected mlpk/mlpk plants from MF2 lines.

Table S1 Primers

	Primer Name	
SP11-S^{8}	S8SP11-F	5'-CTAATCTGATGAAGCGGTGC-3'
	S8SP11-R	GTAAGTGACTTTTGGAATGAATAG
SP11-S^{9}	S9SP11-F	AGTCATGTTCAAGAAGTGGA
	S9SP11-R	ACAACTGATACATTTGCATTGA
SP11-S^{52}	S52SP11-F	CTCTTTTCTGAATCATGAAATCCG
	S52SP11-R	AGAAAAGAACAGCTGATACTTTTAC
SP11-S^{60}	S60SP11-F	ATGAAAGGTGTACGAAACATC
	S60SP11-R	GGATGTTTCGTTGATCAATTATG
MLPK	wtMLPK-F	GCTCTTGGTTGTGCAAATG
	mMLPK-R	GCTCTTGGTTGTGCAAATC
	wtmMLPK-R	AGACATCTGAACACTTGAGTAGC
V-ATPase	BraVATPase-F	GCGAGGGCATGACTCGTAAA
	BraVATPase-R	GCGACTGGAAGATGTTGCGAGT
