# Supplementary material for: Involvement of MLPK Pathway in Intraspecies Unilateral Incompatibility Regulated by a Single Locus With Stigma and Pollen Factors
Source: G3 (Bethesda). 2013 Apr 1;3(4):719–26. doi: 10.1534/g3.113.005892 (PMC3618358; doi:10.1534/g3.113.005892)
Supplement: Supporting Information [file supp_g3.113.005892_FigureS1.pdf]

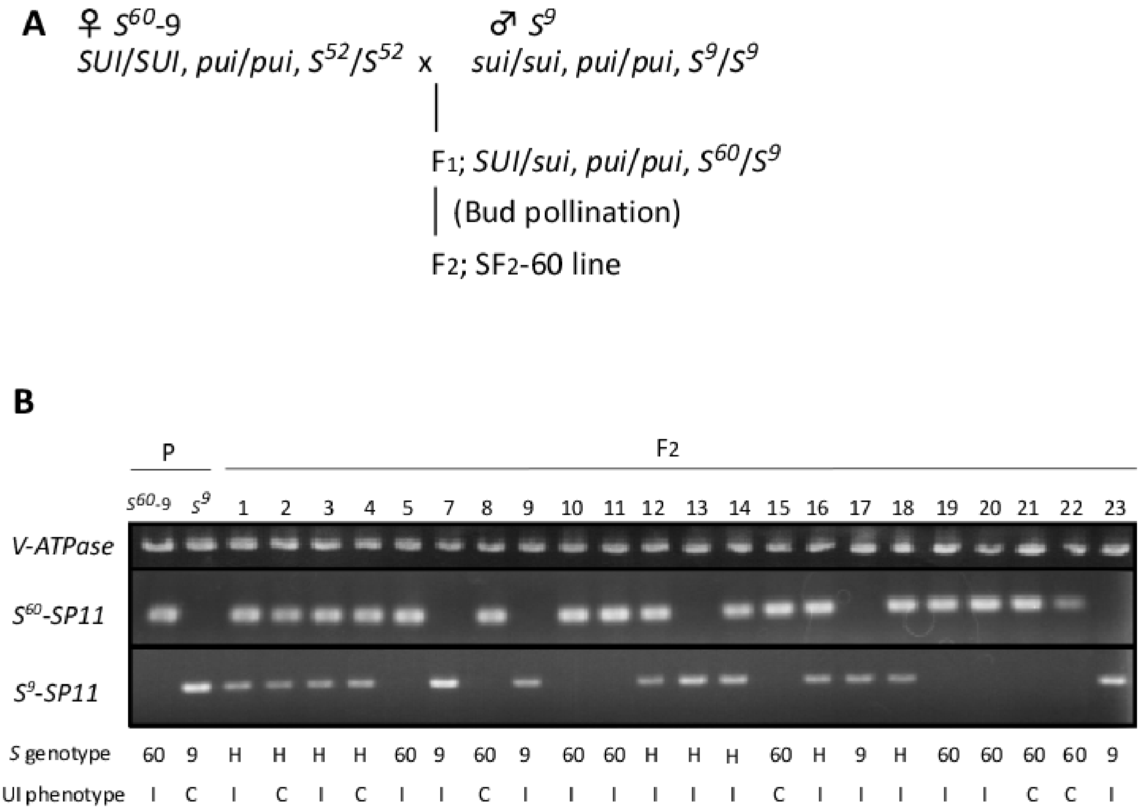

Figure S1 Segregation analysis of SF_2-60.

(A) Crossing scheme used to develop the SF_2-60 segregation line. (B) Genomic DNA isolated from parental (P) plants homozygous for either the S^{60}-9 or S^{9}, and SF_2-60 progeny plants (F_2) and their *SP11* were amplified using their *S*-genotype specific primer. The SUI phenotype of the stigma of each plant to S^{40}t pollen was determined by pollination tests. The *S* genotype and UI phenotype are shown below each lane: 60, S^{60}-homozygote; 9, S^{9}-homozygote; H, S^{60}/S^{9}-heterozygote; C, compatible to S^{40}t pollen (non-UI); I, incompatible to S^{24}t (UI). *V-ATPase* gene was amplified as a positive control.
