# Supplementary material for: Involvement of MLPK Pathway in Intraspecies Unilateral Incompatibility Regulated by a Single Locus With Stigma and Pollen Factors
Source: G3 (Bethesda). 2013 Apr 1;3(4):719–26. doi: 10.1534/g3.113.005892 (PMC3618358; doi:10.1534/g3.113.005892)
Supplement: Supporting Information [file supp_g3.113.005892_TableS1.pdf]

Table S1 Primers

	Primer Name	
SP11-S^8	S8SP11-F	5'-CTAATCTGATGAAGCGGTGC-3'
	S8SP11-R	GTAAGTGACTTTTGGAATGAATAG
SP11-S^9	S9SP11-F	AGTCATGTTCAAGAAGTGGA
	S9SP11-R	ACAACTGATACATTTGCATTGA
SP11-S^{52}	S52SP11-F	CTCTTTTCTGAATCATGAAATCCG
	S52SP11-R	AGAAAAGAACAGCTGATACTTTTAC
SP11-S^{60}	S60SP11-F	ATGAAAGGTGTACGAAACATC
	S60SP11-R	GGATGTTTCGTTGATCAATTATG
MLPK	wtMLPK-F	GCTCTTGGTTGTGCAAATG
	mMLPK-R	GCTCTTGGTTGTGCAAATC
	wtmMLPK-R	AGACATCTGAACACTTGAGTAGC
V-ATPase	BraVATPase-F	GCGAGGGCATGACTCGTAAA
	BraVATPase-R	GCGACTGGAAGATGTTGCGAGT
